# Wernicke’s encephalopathy as a presentation of severe thiamine deficiency after cardiac valve surgery: A case report and narrative review

**Published:** 2019-04-04

**Authors:** Payam Sarraf, Dina Motamedi, Arman Habibi, Sama Bitarafan

**Affiliations:** 1 Iranian Center of Neurological Research, Neuroscience Institute, Tehran University of Medical Sciences, Tehran, Iran; 2 Imam Khomeini Hospital, Tehran University of Medical Sciences, Tehran, Iran; 3 Department of Neurology, School of Medicine, Kurdistan University of Medical Sciences, Sanandaj, Iran

**Keywords:** Cardiac Surgery, Wernicke Encephalopathy, Thiamine Deficiency

Thiamine has a key role in the regulation of cell metabolism. Thiamine is considered as an important co-factor for crucial enzymes in carbohydrate metabolism to preserve brain health. Severe deficiency of thiamine is impaired to mitochondrial function and metabolic system in brain. The mechanisms are disruption of blood brain barrier, inducing inflammation, and degeneration in the neurological system probably due to increased oxidative stress and lactic acidosis. Its deficiency induces types of beriberi, characterized by neuropathy, Wernicke’s encephalopathy (WE), and heart failure. Although delay in the treatment of thiamine deficiency can be life-threatening, early supplementation shows a quick beneficial effect. Thiamine deficiency has reported among the critically ill patients with major stress, especially those who were under surgery, and receive parenteral nutrition.^1^

Many patients with neurological presentations of thiamine deficiency have missed because there is often no history of alcohol abuse or malnutrition accompanied by a lack of reliable diagnostic test. Diagnosis of WE is according to the classical triad of acute confusion, ophthalmoplegia and ataxia. Misdiagnosis of WE can lead to a chronic mental dysfunction known as Korsakoff’s syndrome in 85% of survivors.^2^

The stress of major surgeries such as coronary artery bypass grafts or gastric surgeries with malabsorption conditions may deplete some essential biochemical substrates such as thiamine, and consequently induce WE in a chronic condition.^3^^,^^4^

A 49-year-old woman with a history of diabetes mellitus, hypothyroidism, mitral valve prolapse, and surgery about 18 years ago was referred to our emergency department.

The chief complaint was severe functional class 3 dyspnea and transesophageal echocardiography revealed malfunction of mechanical mitral valve with superimposed thrombosis. The patient was prepared for redo mitral and tricuspid valves regurgitation operation, and the procedure took about 6 hours. According to routine postoperative examination, patient was extubated, and transferred to postoperative room in 6 hours with only slight drowsiness; but she was intubated again in the second postoperative day because of poor respiratory function. The cardio-surgeon requested an emergency neurological consulting. 

At the first neurological evaluation, she was lethargic and only responded to painful stimuli by opening her eyes for about 10 seconds, shouting and aggressive aimless projection of her limbs with little obedience meanwhile, and then became calm again without stimulus. Right pupil was in mid-position and mydriatic about 7 mm, without light response, but left pupil was normal. No myogenic ophthalmoplegia and ptosis were detected. Fundoscopic examination was normal in both sides. Neurological examination disclosed ocular movement abnormalities. There was neither peripheral nervous system nor metabolic-related signs or symptoms. 

There was no significant point in computerized tomography (CT) scan. Therefore, brain magnetic resonance imaging (MRI) was requested. MRI revealed bilateral periventricular hyper-intensity, compatible with microvascular changes, along with bilateral periaqueductal and thalamic pulvinar nuclei foci with restriction on diffusion-weighted imaging and hyper-signal on T2-wieghted sequences (Figure 1). 

At first according to clinical signs and MRI features, differential diagnoses such as cardio-embolic stroke or watershed infarcts due to hypoxic-ischemic insult, and electrolyte abnormalities were considered. Serum thiamine measurement test was not available. However, because of the incidence of post-surgery WE, WE due to thiamine deficiency was more likely. 

**Figure 1:**
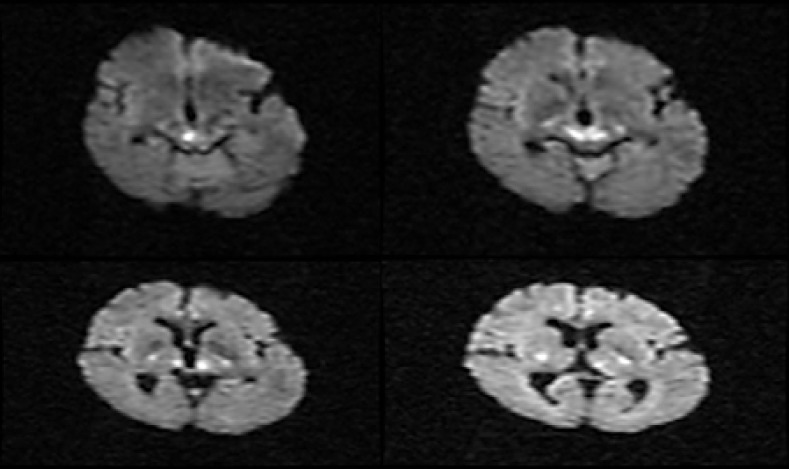
Evidence of restriction in periaqueductal and thalamic region

Studies have demonstrated the efficacy and safety of parenteral thiamine 200-500 mg, three times a day should be given for 3-5 days, followed by oral thiamine 250-1000 mg/day in WE.^5^ So, a full course of intravenous (IV) thiamine therapy was prescribed for this patient (200 mg, 3 times a day for 3 days). The patient situation was incredible in about 2-days-recovery. She aroused by calling her name with a proper response to simple questions such as her age, her daughter’s name, but not to the date or about the reason of being administrated. Treatment was continued with maintenance dose of oral thiamine (100 mg daily). At the fifth day, she was completely alert and cooperative with little slurred speech, ataxic limb movements, and gait ataxia without sensory symptoms. The patient responded to the treatment and tolerated well.

In conclusion, the study of the mentioned case indicates the importance of considering thiamine depletion in patients with WE, especially during severe stress such as heart surgery, while early treatment can prevent WE's severe sequels which can be disabling or even life-threatening.

## Notes:

**How to cite this article:** Sarraf P, Motamedi D, Habibi A, Bitarafan S. Wernicke’s encephalopathy as a presentation of severe thiamine deficiency after cardiac valve surgery: A case report and narrative review. Iran J Neurol 2019; 18(2): 85-6.
